# The association between normal-range admission potassium levels in Israeli patients with acute coronary syndrome and early and late outcomes

**DOI:** 10.1097/MD.0000000000003778

**Published:** 2016-06-10

**Authors:** Gadi Shlomai, Anat Berkovitch, Shiran Pinchevski-Kadir, Gil Bornstein, Avshalom Leibowitz, Ilan Goldenberg, Ehud Grossman

**Affiliations:** a Department of Internal Medicine D and Hypertension Unit; b The Dr Pinchas Borenstein Talpiot Medical Leadership Program 2013; c Heart Institute and the Neufeld Cardiac Research Institute, Leviev Heart Center, the Chaim Sheba Medical Center, Tel-Hashomer, Sackler Faculty of Medicine, Tel-Aviv University, Tel-Aviv, Israel.

**Keywords:** acute coronary syndrome, admission serum potassium, ventricular arrhythmia

## Abstract

Abnormal serum potassium levels are associated with an increased risk of ventricular arrhythmias and mortality in patients with acute myocardial infarction (AMI). The aim of the present study was to evaluate whether different levels of serum potassium, within the normal range, are associated with worse outcomes. The present study comprised 1277 patients with AMI and normal-range admission potassium levels (3.5–5.2 mEq/L), who were enrolled and prospectively followed up in the Acute Coronary Syndrome Israeli Survey between 2010 and 2013. Patients were divided into 4 quartiles based on admission potassium levels; “normal-low” (K ≥ 3.5 and K ≤ 3.9), “normal-moderate” (K > 3.9 and K ≤ 4.18), “normal-high” (K > 4.18 and K ≤ 4.45), and “normal-very high” (K > 4.45 and K ≤ 5.2). We analyzed the association between admission serum potassium levels and 7 days in-hospital complication rates, and 30-day and 1-year all-cause mortality rates. Patients with “normal-very high” potassium displayed increased frequency of baseline clinical risk factors and experienced a higher rate of acute kidney injury during hospitalization compared with the “normal-low” group (7.7% vs 2.4%; *P* = 0.002). However, the rate of in-hospital ventricular arrhythmias was similar across the range of admission potassium levels (overall *P* = 0.26), Multivariate analysis showed that compared with “low-normal” potassium values, patients with “normal-very high” potassium levels experienced increased risk for 30-days (adjusted hazard ratio 2.88, 95% confidence interval 1.05–7.87, *P* = 0.039) and 1-year all-cause mortality (adjusted hazard ratio 1.98, 95% confidence interval 1.05–3.75, *P* = 0.034). In patients admitted with AMI, admission serum potassium levels of 4.45 to 5.2 mEq/L are not associated with in-hospital ventricular arrhythmias, but are associated with increased short and long-term mortality.

## 1 Introduction

Serum potassium levels play a major role in the outcome of cardiovascular (CV) events.^[1]^ Changes in intracellular and extracellular potassium levels modify the electrophysiological properties of the resting membrane potential in cardiac cells and subsequently influence myocardial impulses generation and conduction.^[2,3]^ Serum potassium levels are maintained between 3.5 and 5.2 mEq/L by renal excretion, and shift between intracellular and extracellular fluid compartments.^[3]^ In the early phases of acute myocardial infarction (AMI), the sympathetic nervous system is activated, as reflected by elevated levels of plasma catecholamines^[4]^ and modulation of β adrenergic receptor signaling.^[5]^ This activation leads to intracellular influx of potassium and decrease in serum potassium levels.^[6]^ Low potassium levels have been shown to increase the automaticity and excitability of myocardial cells, leading to the propensity for ventricular arrhythmias.^[1]^

Several studies have previously illustrated the prognosticator role of profoundly low admission serum potassium, usually below 3.5 mEq/L, and the risk of in-hospital ventricular arrhythmias, among patients with AMI.^[4,7–14]^ In a recent large retrospective study, the authors have found a U-shaped relationship between admission serum potassium levels and in-hospital morbidity and mortality among AMI patients.^[15]^ However, Madias et al^[16]^ did not find admission potassium levels to be a predictor of increased morbidity and mortality among patients with AMI.

Data regarding the association between admission serum potassium levels, within the normal range, and morbidity and mortality outcomes among AMI patients are still lacking. Therefore, in this study we evaluated the association between admission serum potassium levels, within the normal range, and early and late outcomes among AMI patients enrolled in the Acute Coronary Syndrome Israeli Survey (ACSIS).

## 2 Methods

### 2.1 Study design

Acute Coronary Syndrome Israeli Survey is a biannual national ACS survey that prospectively collects data from all patients admitted with ACS during a 2-month period from each of the coronary care units and cardiology wards operating in Israel. A detailed report of the survey design and methods has previously been published.^[17]^ The study protocol was approved by the local institutional review board in accordance with the Declaration of Helsinki, and a waiver of consent has been granted to the investigators.

### 2.2 Patients

The complete ACSIS 2010 to 2013 survey database includes 1413 subjects. Only subjects with serum potassium levels within the normal values (3.5–5.2 mEq/L) at presentation were included in the current study. Subjects were excluded if their potassium level was lower than 3.5 mEq/L (n = 76) or higher than 5.2 mEq/L (n = 60). Thus, the final study sample comprised 1277 subjects.

### 2.3 Data collection

Demographic, associated diseases, medical therapy, clinical presentation, admission blood pressure (BP) and heart rate values, electrocardiography, and baseline laboratory results were recorded on prespecified forms for all patients admitted with a diagnosis of ACS.

In-hospital and 30-day outcomes data were ascertained by hospital chart review, telephone contact, and clinical follow-up data. Out-of-hospital 1-year mortality data were ascertained through the use of the Israeli National Population Registry. Data checks for completeness and consistency were based on computerized data queries issued at the coordinating center of the Israeli Association for Cardiovascular Trials and were completed from the medical reports attached to the form of each patient.

### 2.4 Definitions and end points

Hypertension was defined when a documented diagnosis was reported or when the patient was chronically prescribed antihypertensive medications. Diabetes mellitus (DM) was defined when fasting serum glucose was >126 mg/dL (7.0 mmol/L) on 2 separate readings, a history of DM was reported, or when insulin or oral hypoglycemic were taken. Smoking status was determined according to the questionnaire. Chronic renal failure was defined when a documented medical diagnosis was reported or when the calculated glomerular filtration rate was less than 60 mL/min. Congestive heart failure was defined when a documented medical diagnosis was reported or when the ejection fraction before admission was less than 40%. Dyslipidemia was defined by history, levels of cholesterol on admission and or use of lipids lowering medications.

Patients were divided into 4 quartiles based on admission potassium values: “normal-low” (K ≥ 3.5 and K ≤ 3.9 mEq/L) (n = 336), “normal-moderate” (K > 3.9 and K ≤ 4.18 mEq/L) (n = 305), “normal-high” (K > 4.18 and K ≤ 4.45 mEq/L) (n = 321), and “normal-very high” (K > 4.45 and K ≤ 5.2 mEq/L) (n = 315).

End points included 30-day and 1-year all-cause mortality.

### 2.5 Statistical analysis

For the univariate analysis, percentages were calculated for categorical variables and means with standard deviations for continuous variables. The linear-by-linear association chi-square test in case of categorical variables and the 1-way analysis of variance (ANOVA) test in case of continuous variables.

For the study's 2 outcomes (30-day and 1-year mortality), we depicted Kaplan–Meier survival curves by potassium categories. The curves were statistically compared using the log-rank test for overall.

Cox proportional-hazards models were used for the 30-day and 1-year mortality outcomes. The models were adjusted to prespecified baseline parameters; age, sex, glomerular filtration rate, hypertension, dyslipidemia, and DM.

All *P*-value calculations were 2-tailed and were considered statistically significant if their value was <0.05. The statistical analyses were performed with IBM SPSS version 22.0 (Chicago, IL).

## 3 Results

The final study population comprised 1277 individuals, mean age was 64 ± 13 years and 991 (78%) were males. Mean follow-up was 482 ± 139 days. Compared with patients with “low-normal” potassium levels, patients with “normal-very high” potassium levels were older and had significantly higher rates of renal failure, DM, and previous myocardial infarctions (Table 1). Patients with “normal-very high” potassium levels had less ST-elevation myocardial infarction and had less reperfusion therapy (Table 1). Preadmission treatment was the same in all groups, and the use of beta-blockers, blockers of the rennin-angiotensin system, and diuretics increased during treatment to the same extent in all groups (Table 2).

**Table 1:**
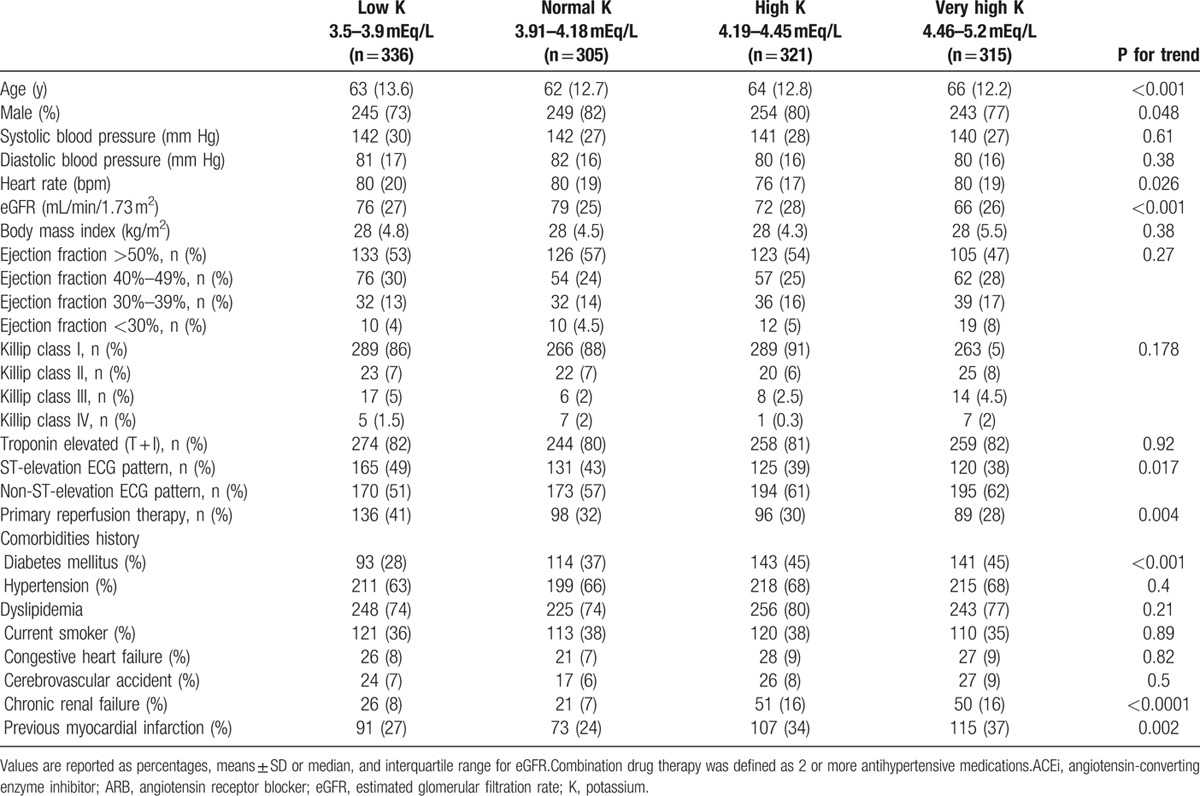
Baseline and in-hospital characteristics of patients with acute coronary syndrome by admission systolic blood pressure category

**Table 2:**
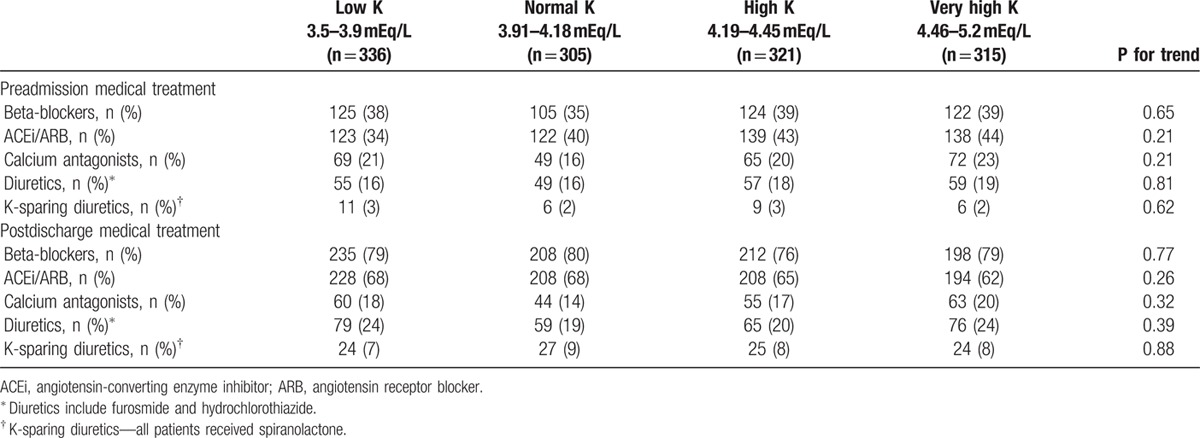
Medical treatment before admission and post discharge

### 3.1 In-hospital complications according to prespecified potassium groups

**Figure 1:**
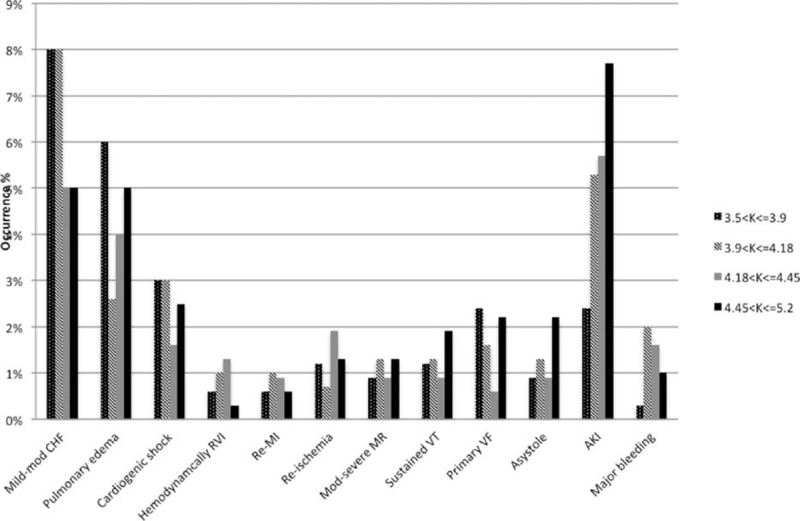
Seven days in-hospital complication rates. Figure shows complication rates (in percentage) at 7 days according to the prespecified potassium groups. AKI, acute kidney injury; CHF, congestive heart failure; MI, myocardial infarction; MR, mitral regurgitation; RVI, right ventricular infarction; VF, ventricular fibrillation; VT, ventricular tachycardia.

Subjects in all prespecified groups demonstrated similar rates of most in-hospital complications (Fig. 1). Acute kidney injury was significantly more common among the “normal-very high” potassium group compared with the “normal-low admission” potassium group (7.7% vs 2.4%; *P* = 0.002) (Fig. 1).

### 3.2 Short and long-term mortality rates

**Figure 2:**
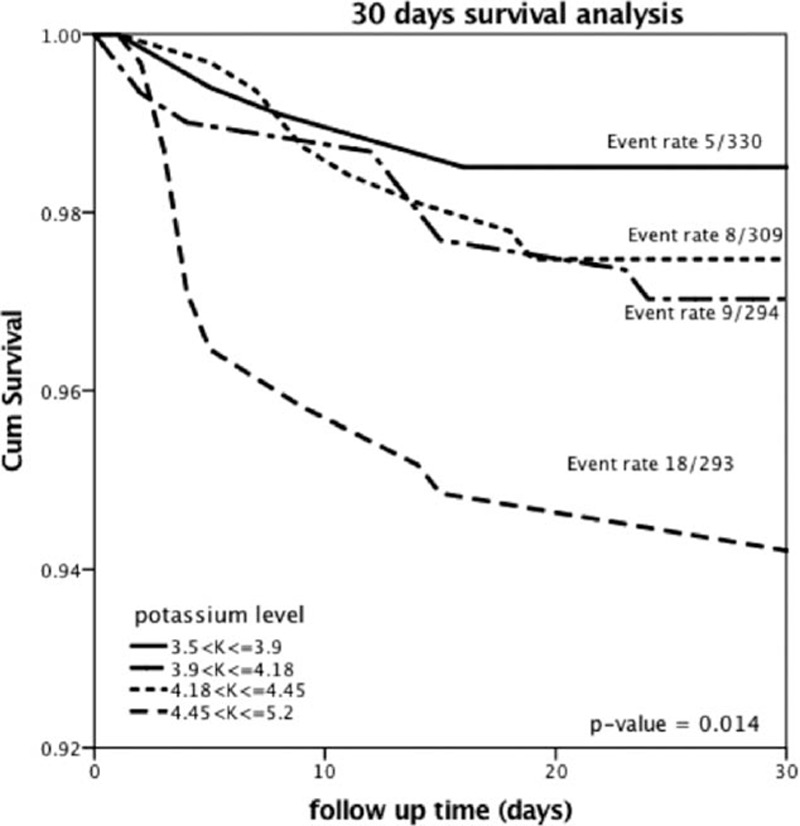
Thirty-day Kaplan–Meier survival analysis. The Kaplan–Meier analysis was used to show mortality probability at 30 days according to the prespecified potassium groups.

Kaplan–Meier survival analysis showed that cumulative probability of all-cause mortality at 30 days was significantly higher among patients in the “normal-very high” potassium group (6%) as compared with the “normal-low” group (1%), the “normal-moderate” group (3%), and the “normal-high” group (3%) (log-rank *P* value = 0.01 for the overall comparison among the 4 groups during 30-days of follow-up; Fig. 2).

**Figure 3:**
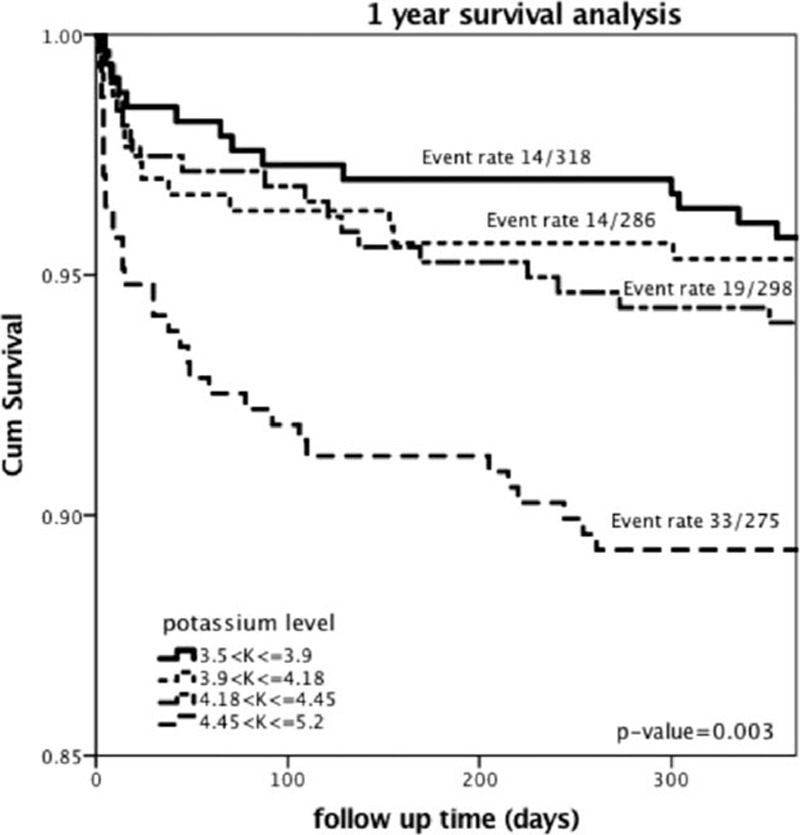
One-year Kaplan–Meier survival analysis. The Kaplan–Meier analysis was used to show mortality probability at 1 year according to the prespecified potassium groups.

Similarly, when the end point of 1-year mortality was assessed, Kaplan–Meier survival analysis showed that patients in the “normal-very high” potassium group experienced significantly higher 1-year mortality rates (11%) compared with the “normal-low” group (4%), the “normal-moderate” group (5%), and the “normal-high” group (6%) (log-rank *P* value = 0.003 for the overall comparison among the 4 groups during 30-days of follow-up; Fig. 3).

In the multivariate adjusted regression model, compared with patients with “low-normal” potassium levels, patients with “normal-very high” potassium levels had increased all-cause mortality at both 30 days and 1 year (Table 3) (hazard ratio [HR] 2.88, 95% confidence interval [CI] 1.05–7.87, *P* = 0.039; and HR 1.98, 95% CI 1.05–3.75, *P* = 0.034, respectively).

**Table 3:**
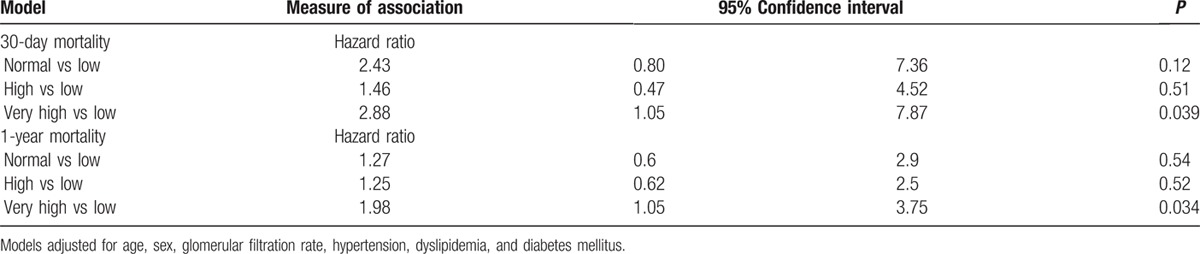
Multivariate Cox regression analysis

## 4 Discussion

We have shown that compared with ACS patients with “low-normal” admission potassium levels, patients with “normal-very high” admission potassium levels had increased all-cause mortality at both 30 days and 1 year. However, we did not find significant differences between groups for in-hospital complication rates, including ventricular arrhythmias. To the best of our knowledge, no studies have previously described the association between normal-range admission potassium levels and mortality outcomes among AMI patients.

Several studies have previously described the relationship of either profoundly low^[4,7–14]^ or extremely high^[15]^ admission potassium levels and increased in-hospital morbidity and mortality, particularly secondary to ventricular arrhythmias. However, most of these studies were relatively small (less than 1000 patients) and were conducted before the era of routine use of beta-blockers and early reperfusion therapies,^[4,7–12]^ which have shown to reduce the incidence of postinfarction mortality and sudden cardiac death.^[10,16]^ Interestingly, Choi et al^[18]^ have recently demonstrated that a U-shaped relation exists between mean serum potassium levels and 3-year mortality among patients with AMI. In particular, mortality was higher in patients with mean potassium levels above 4.5 mEq/L.^[18]^

The β_1_ adrenergic receptor signaling abnormalities contribute to the development of arrhythmias.^[19]^ During AMI, β_1_ adrenergic receptor signaling is associated with cardiac hypertrophy, ventricular remodelling, and subsequently development of ventricular arrhythmias.^[19]^ Inversely, β_2_ adrenergic receptor activation has been found to attenuate cardiac remodeling in myocardial ischemic insult, thereby preserving cardiac function after AMI.^[20,21]^ The occurrence rate of the majority of in-hospital complications, including ventricular arrhythmias, was found to be similar across all prespecified groups. This finding is of particular interest compared with previous reports, which emphasized the association of potassium imbalance upon admission and the increased risk for in-hospital ventricular arrhythmias.^[4,7–11,13–15]^ This discrepancy might be explained by the fact that in previous studies, in-hospital morbidity and mortality were only significantly associated with extremely abnormal admission potassium levels, either profoundly low or high, whereas in our analysis, we have only included patients with normal-range potassium levels. Notably, Choi et al have also recently shown that there was no relation between mean serum potassium levels and the occurrence of ventricular arrhythmias.^[18]^

The prognosticator role of normal range admission potassium levels among patients with AMI have been described in the past. Higher potassium levels (≥4.3 mEq/L) were shown to be an independent risk factor for target lesion revascularization among AMI patients.^[22]^ In addition, higher admission potassium levels (≥4.3 mEq/L) were also found to be associated with a larger scintigraphic infarct size in patients with ST-elevation myocardial infarction.^[23]^ These findings suggest that higher potassium levels, although in the normal range, may be associated with complications that are not necessarily related to immediate changes in the electrophysiological properties of the myocardium, and thus can be associated with late morbidity and mortality. For example, infarct size has been shown to be one of the main determinants of outcome in patients with AMI, even after 6 months.^[24]^ These findings provide a plausible explanation to our results, which demonstrated increased 30-day and 1-year mortality among patients with potassium values of 4.45 to 5.2 mEq/L, yet no increase of in-hospital complication rates, such as ventricular arrhythmias. It is possible that whereas normal range potassium levels are not associated with immediate detrimental conductive aberrancies, they are related to other structural and functional abnormalities which manifest later on, even after several months. Elevated admission potassium levels, even within the normal range, may be an indicator of severe sickness, thereby explaining the increased mortality. We indeed observed a higher rate of DM and renal failure in those with elevated admission potassium levels. Hyperkalemia occurs more frequently in patients with DM compared with nondiabetic patients.^[25]^. The syndrome of hyporeninemic hypoaldosteronism (HH) is considered the most important causal factor for chronic hyperkalemia in DM patients, but other proposed mechanisms include hyperosmolality, insulin resistance or deficiency, and potassium-sparing antihypertensive medications.^[26]^ Notably, HH has been associated with increased risk of microvascular complications including diabetic nephropathy and neuropathy.^[27]^ Therefore, the increased mortality rates in patients with higher admission potassium levels observed in our study might be confounded by pre-existing DM and renal failure. However, the increase short and long-term mortality in these patients was significant even after adjustment for various comorbidities, including DM and renal function.

Our study has several limitations. It is an observational study and is restricted to Israeli adults, and as such it is unclear whether these findings could be generalized to other populations. We have only data on all-cause mortality and we do not know whether the increased mortality in patients with “normal-very high” admission potassium levels was related to CV causes. In addition, we do not have information regarding the long-term treatment of patients after getting discharged from the hospital, which could affect their survival and hospitalizations. Nevertheless, our analysis included a large number of patients from all intensive cardiac care units and cardiology wards in all public hospitals in Israel. The similar associations of the admission potassium level categories with 2 different end points strengthen the validity of our findings.

In conclusion, our data suggest that admission potassium levels, even when in the normal range, might play a significant role in the risk stratification process of patients presenting with AMI.

## References

[1] Al-Quthami AH Udelson JE What is the “goal” serum potassium level in acute myocardial infarction? *Am J Kidney Dis* 2012; 60:517–520. 22742865 10.1053/j.ajkd.2012.05.011

[2] El-Sherif N Turitto G Electrolyte disorders and arrhythmogenesis. *Cardiol J* 2011; 18:233–245. 21660912

[3] Macdonald JE Struthers AD What is the optimal serum potassium level in cardiovascular patients? *J Am Coll Cardiol* 2004; 43:155–161. 14736430 10.1016/j.jacc.2003.06.021

[4] Clausen TG Brocks K Ibsen H Hypokalemia and ventricular arrhythmias in acute myocardial infarction. *Acta Med Scand* 1988; 224:531–537. 3207065 10.1111/j.0954-6820.1988.tb19623.x

[5] Santulli G Campanile A Spinelli L G protein-coupled receptor kinase 2 in patients with acute myocardial infarction. *Am J Cardiol* 2011; 107:1125–1130. 21296320 10.1016/j.amjcard.2010.12.006

[6] Brown MJ Hypokalemia from beta 2-receptor stimulation by circulating epinephrine. *Am J Cardiol* 1985; 56:3d–9d. 10.1016/0002-9149(85)91107-5 2863972

[7] Friedensohn A Faibel HE Bairey O Malignant arrhythmias in relation to values of serum potassium in patients with acute myocardial infarction. *Int J Cardiol* 1991; 32:331–338. 1686433 10.1016/0167-5273(91)90295-z

[8] Hulting J In-hospital ventricular fibrillation and its relation to serum potassium. *Acta Med Scand Suppl* 1981; 647:109–116. 6942634 10.1111/j.0954-6820.1981.tb02646.x

[9] Kafka H Langevin L Armstrong PW Serum magnesium and potassium in acute myocardial infarction. Influence on ventricular arrhythmias. *Arch Intern Med* 1987; 147:465–469. 3827422

[10] Nordrehaug JE Johannessen KA von der Lippe G Serum potassium concentration as a risk factor of ventricular arrhythmias early in acute myocardial infarction. *Circulation* 1985; 71:645–649. 3971535 10.1161/01.cir.71.4.645

[11] Nordrehaug JE von der Lippe G Hypokalaemia and ventricular fibrillation in acute myocardial infarction. *Br Heart J* 1983; 50:525–529. 6651995 10.1136/hrt.50.6.525 PMC481454

[12] Peng Y Huang FY Liu W Relation between admission serum potassium levels and long-term mortality in acute coronary syndrome. *Intern Emerg Med* 2015; 10:927–935. 25986480 10.1007/s11739-015-1253-1

[13] Solomon RJ Cole AG Importance of potassium in patients with acute myocardial infarction. *Acta Med Scand Suppl* 1981; 647:87–93. 6942645 10.1111/j.0954-6820.1981.tb02643.x

[14] Su J Fu X Tian Y Additional predictive value of serum potassium to Thrombolysis In Myocardial Infarction risk score for early malignant ventricular arrhythmias in patients with acute myocardial infarction. *Am J Emerg Med* 2012; 30:1089–1094. 22035586 10.1016/j.ajem.2011.07.009

[15] Goyal A Spertus JA Gosch K Serum potassium levels and mortality in acute myocardial infarction. *JAMA* 2012; 307:157–164. 22235086 10.1001/jama.2011.1967

[16] Madias JE Shah B Chintalapally G Admission serum potassium in patients with acute myocardial infarction: its correlates and value as a determinant of in-hospital outcome. *Chest* 2000; 118:904–913. 11035655 10.1378/chest.118.4.904

[17] Behar S Battler A Porath A A prospective national survey of management and clinical outcome of acute myocardial infarction in Israel, 2000. *Israel Med Assoc J* 2003; 5:249–254. 14509128

[18] Choi JS Kim YA Kim HY Relation of serum potassium level to long-term outcomes in patients with acute myocardial infarction. *Am J Cardiol* 2014; 113:1285–1290. 24560065 10.1016/j.amjcard.2014.01.402

[19] Bhushan S Kondo K Predmore BL Selective beta2-adrenoreceptor stimulation attenuates myocardial cell death and preserves cardiac function after ischemia-reperfusion injury. *Arterioscl Thromb Vasc Biol* 2012; 32:1865–1874. 22652602 10.1161/ATVBAHA.112.251769 PMC3401356

[20] Bernstein D Fajardo G Zhao M Differential cardioprotective/cardiotoxic effects mediated by beta-adrenergic receptor subtypes. *Am J Physiol Heart Circulat Physiol* 2005; 289:H2441–2449. 10.1152/ajpheart.00005.2005 16040722

[21] Zhu WZ Zheng M Koch WJ Dual modulation of cell survival and cell death by beta(2)-adrenergic signaling in adult mouse cardiac myocytes. *Proc Natl Acad Sci USA* 2001; 98:1607–1612. 11171998 10.1073/pnas.98.4.1607 PMC29304

[22] Honda T Fujimoto K Miyao Y Potassium concentration on admission is an independent risk factor for target lesion revascularization in acute myocardial infarction. *Sci World J* 2014; 2014:946803. 10.1155/2014/946803 PMC3913530 24523655

[23] Roos M Ndrepepa G Baumann M Serum potassium levels on admission and infarct size in patients with acute myocardial infarction. *Clin Chim Acta* 2009; 409:46–51. 19720056 10.1016/j.cca.2009.08.014

[24] Burns RJ Gibbons RJ Yi Q The relationships of left ventricular ejection fraction, end-systolic volume index and infarct size to six-month mortality after hospital discharge following myocardial infarction treated by thrombolysis. *J Am Coll Cardiol* 2002; 39:30–36. 11755283 10.1016/s0735-1097(01)01711-9

[25] Uribarri J Oh MS Carroll HJ Hyperkalemia in diabetes mellitus. *J Diab Complicat* 1990; 4:3–7. 10.1016/0891-6632(90)90057-c 2141843

[26] Karet FE Mechanisms in hyperkalemic renal tubular acidosis. *J Am Soc Nephrol* 2009; 20:251–254. 19193780 10.1681/ASN.2008020166

[27] Sousa AG Cabral JV El-Feghaly WB Hyporeninemic hypoaldosteronism and diabetes mellitus: pathophysiology assumptions, clinical aspects and implications for management. *World J Diab* 2016; 7:101–111. 10.4239/wjd.v7.i5.101 PMC4781902 26981183

